# On the mass spectrometric fragmentations of the bacterial sesterterpenes sestermobaraenes A–C

**DOI:** 10.3762/bjoc.16.231

**Published:** 2020-11-19

**Authors:** Anwei Hou, Jeroen S Dickschat

**Affiliations:** 1 Kekulé-Institute for Organic Chemistry and Biochemistry, University of Bonn, Gerhard-Domagk-Strasse 1, 53127 Bonn, Germany

**Keywords:** isotopes, mass spectrometry, reaction mechanisms, sesterterpenes, *Streptomyces mobaraensis*

## Abstract

A ^13^C-labelling was introduced into each individual carbon of the recently discovered sestermobaraenes by the enzymatic conversion of the correspondingly ^13^C-labelled isoprenyl diphosphate precursors with the sestermobaraene synthase from *Streptomyces mobaraensis*. The main compounds sestermobaraenes A, B, and C were analysed by gas chromatography–mass spectrometry (GC–MS), allowing for a deep mechanistic investigation of the electron impact mass spectrometry (EIMS) fragmentation reactions of these sesterterpene hydrocarbons.

## Introduction

**Figure 1:**
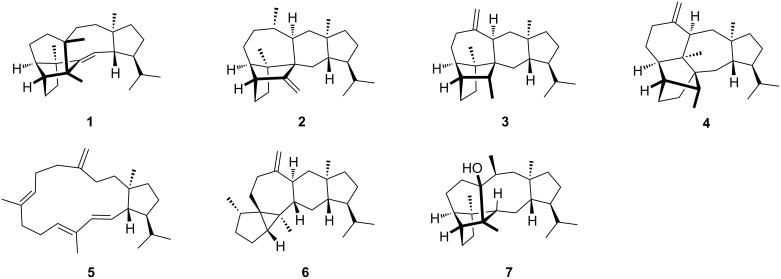
The structures of the bacterial sesterterpenes sestermobaraenes A–F (**1**–**6**) and sestermobaraol (**7**) from *Streptomyces mobaraensis*.

The sestermobaraenes A–F (**1**–**6**) and sestermobaraol (**7**) are a series of bacterial sesterterpenes that were recently discovered by us from the actinomycete *Streptomyces mobaraensis* through a genome mining approach (Figure 1) [1]. All seven compounds are produced by a canonical terpene synthase, representing the first reported sesterterpene synthase of the classical type I from bacteria, that is characterised by an aspartate-rich motif (DDXXD) and an NSE triad (NDLXSXXXE) for binding of a trinuclear Mg^2+^ cluster [2–3]. The Mg^2+^ cations in turn bind to the diphosphate moiety of an isoprenoid diphosphate precursor and cause substrate ionisation by a diphosphate abstraction to initiate a cationic cyclisation cascade, leading to structurally highly complex and usually polycyclic terpenes in just one enzymatic transformation. The initially formed products are non-functionalised terpene hydrocarbons or, if the terminal cationic intermediate of the cyclisation cascade is trapped by water, simple alcohols. These volatile compounds can efficiently be trapped by specialised methods including the closed-loop stripping apparatus (CLSA) [4] technique or solid-phase microextraction (SPME) [5–6], and then analysed by gas chromatography–mass spectrometry (GC–MS) [7]. Through these and related techniques the volatiles from many bacteria, fungi, and plants have been investigated [8–10], which provides rapid information about the production of volatile terpenes. This information is particularly useful in the combination with the genome sequences of the producing organism, because it allows to identify interesting candidate genes coding for terpene synthases for further studies by genome mining. A major difficulty in the GC–MS-based identification of terpenes is associated with the high similarity of the mass spectra of structurally related terpenes. For this reason, the unambiguous identification of terpenes requires either the direct comparison to an authentic standard, or, since such a standard is not always available, a very good match of the measured mass spectrum to a library spectrum and of the measured retention index to literature data. Mass spectrometric fragmentations proceed through reactions that are classified as σ-bond cleavages, α-fragmentations, inductive cleavages, McLafferty rearrangements [11], retro-Diels–Alder fragmentations [12–13], and the recently observed unusual radical-induced retro-Cope rearrangement (herein, “retro” indicates that the mass spectrometric reaction proceeds in reverse order of a thermal reaction promoted by the thermal conditions of the gaschromatographic analysis) [14]. The fragmentation reactions of structurally simple compounds such as fatty acid methyl esters have been well investigated by isotopic labelling experiments [15–16] and the knowledge allows for structural predictions based on GC–MS data [17]. The deuterium labelling technique was also applied to other compound classes such as alkylbenzenes and ketones [18–21]. For terpenes, structural proposals can only be made based on the mass spectra for structurally less complicated cases, as was exemplified for the side products of bacterial 2-methylisoborneol synthases [22], but in general the structural complexity of terpenes does not allow for such approaches. Nevertheless, more knowledge about the MS fragmentation reactions of terpenes is desirable, but represents a challenging objective as it is difficult to get access to the isotopically labelled terpenes needed for deep and conclusive insights. The early investigations by Djerassi and co-workers have made use of semisynthetic deuterated terpenes [23–25]. While deuterium can reveal specific hydrogen migrations in the fragmentation reactions, is comparably cheap, and can often easily be introduced, e.g., into C,H-acidic positions, a drawback of deuterium usage lies in possible kinetic isotope effects [21]. Also MS/MS-based techniques have been used to study the fragmentations of terpenes [26–28], but isotopic labelling experiments can give more detailed and conclusive insights. We have recently investigated the MS fragmentation mechanisms of several sesqui- and diterpenes in a series of studies that made use of ^13^C-labelled terpene precursors to systematically introduce single labellings into each individual carbon position by enzymatic synthesis [14,29–32]. Here we report on the MS fragmentation mechanisms for the bacterial compounds sestermobaraenes A, B, and C, representing the first mechanistic study of this kind for sesterterpenes.

## Results and Discussion

### Experimental basis

**Figure 2:**
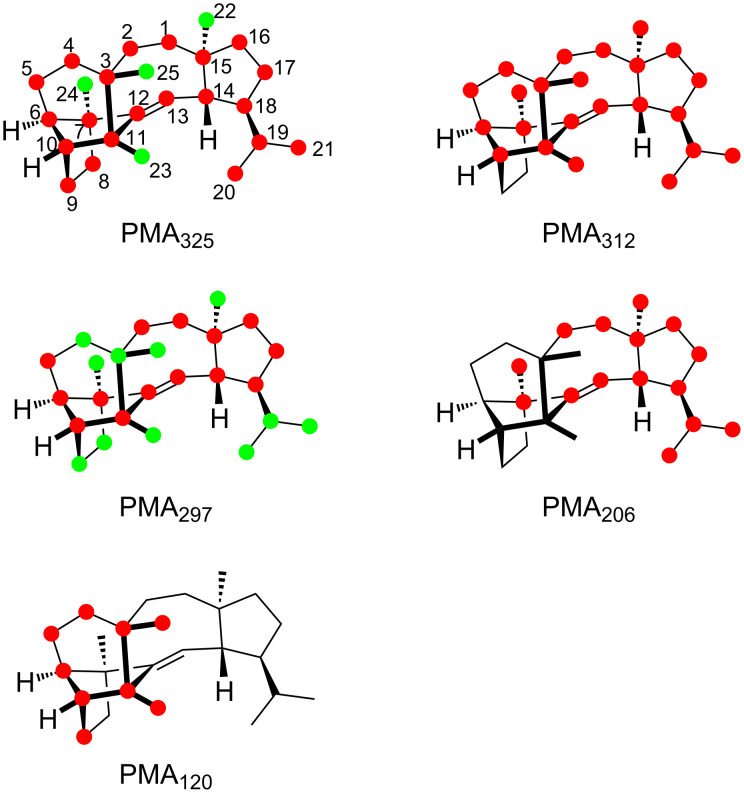
Position-specific mass shift analyses for **1**. Carbons that contribute fully to the formation of a fragment ion are indicated by red dots, partially contributing carbons are marked by green dots, and unlabelled carbons do not contribute and are thus cleaved off by the fragmentation reaction.

**Figure 3:**
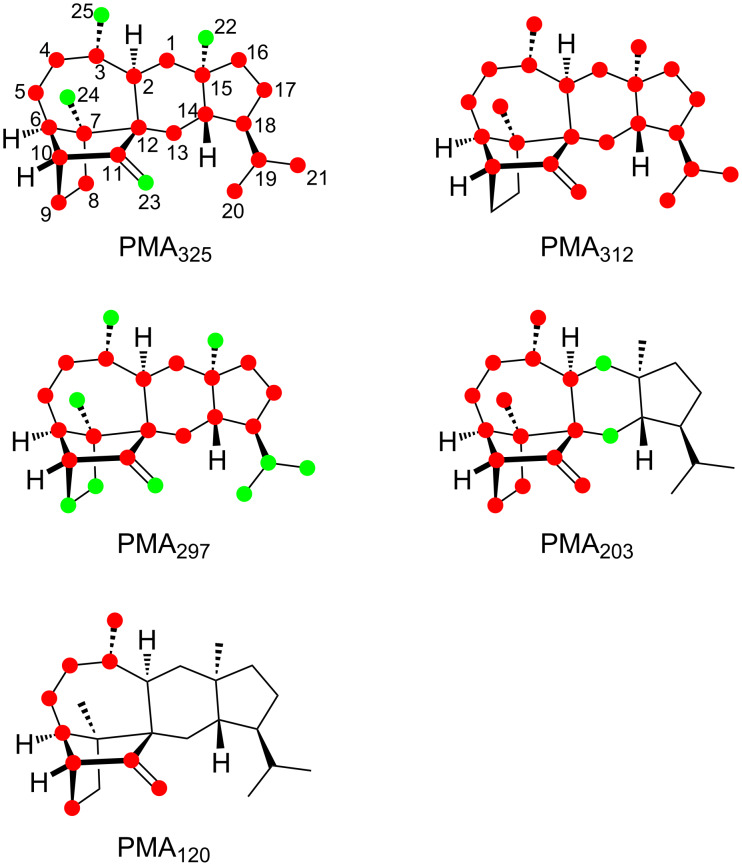
Position-specific mass shift analyses for **2**. The carbons that contribute fully to the formation of a fragment ion are indicated by red dots, partially contributing carbons are marked by green dots, and unlabelled carbons do not contribute and are thus cleaved off by the fragmentation reaction.

**Figure 4:**
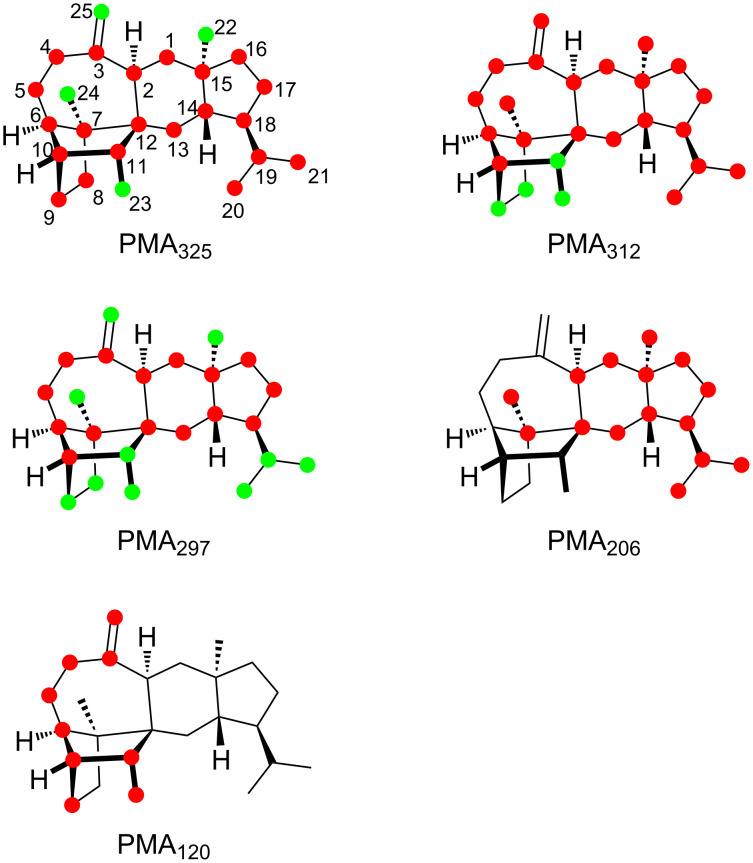
The position-specific mass shift analyses for **3**. Carbons that contribute fully to the formation of a fragment ion are indicated by red dots, partially contributing carbons are marked by green dot, and unlabelled carbons do not contribute and are thus cleaved off by the fragmentation reaction.

The 25 isotopomers of (^13^C)geranylfarnesyl diphosphate (GFPP) were enzymatically prepared from the correspondingly labelled geranyl diphosphate (GPP), farnesyl diphosphate (FPP), geranylgeranyl diphosphate (GGPP), and isopentenyl diphosphate (IPP) with geranylfarnesyl diphosphate synthase (GFPPS) and then converted into mixtures of the sesterterpenes **1**–**7** by the sestermobaraene synthase from *Streptomyces mobaraensis* (SmTS1). All ^13^C-labelled terpene precursors were made available by synthesis in our laboratory in high isotopic purity with ^13^C substitutions of nearly 100% [33–37]. The compound mixtures were subsequently analysed by GC–MS and the mass spectra of the unlabelled compounds **1**–**3** and their 25 singly ^13^C-labelled isotopomers are summarised in Figures S1–S3 in Supporting Information File 1. Investigations on the mass spectrometric fragmentation mechanisms for the minor products **4**–**7** of SmTS1 are not included in this study, because in some cases no high quality mass spectra could be obtained. The mass spectra of the unlabelled compounds show several pronounced signals for fragment ions (*m*/*z*, mass-to-charge ratio). If a signal in a mass spectrum for a particular ^13^C-labelled isotopomer of a compound under investigation is in comparison to the non-labelled compound clearly increased by 1 Da, this means that the labelled carbon fully contributes to the fragment ion. Accordingly, if the signal is clearly not shifted, this means the labelled carbon is not part of the fragment ion. Also cases in between these clear situations exist, namely if a signal in the mass spectrum is a result of two or more fragment ions formed from different parts of the molecule, a labelled carbon may or may not contribute to its formation. A quick overview can be given in a position-specific mass shift analysis for a fragment ion *m*/*z* (PMA_m/z_), in which fully contributing carbons are marked by red dots, partially contributing carbons by green dots, and carbons that do not contribute remain without a mark (Figures 2–4, vide infra). Because usually multiple fragmentation reactions lead to the formation of the ions observed in the low molecular weight region, their formation will not be discussed (an exception is the base peak at *m*/*z* = 120 for all three compounds). The method also finds its limitations for fragment ions buried within a group of peaks. Such fragment ions will not be discussed in this work.

### Fragmentation mechanisms for sestermobaraene A (**1**)

The position-specific mass shift analyses (Figure 2) for several prominent fragment ions observed in the mass spectrum of sestermobaraene A (**1**) are based on a comparison of the mass spectrum of the unlabelled compound **1** to the mass spectra of the 25 isotopomers of (^13^C)-**1** (Figure S1, Supporting Information File 1). As can be concluded from these analyses, the fragment ions observed at *m*/*z* = 312, *m*/*z* = 206, and the base peak at *m*/*z* = 120 are formed by a loss of a clearly defined portion of **1**, while the fragment ions at *m*/*z* = 325 and *m*/*z* = 297 arise through various reactions with losses of different portions of the molecule that can, however, still be rationalised. For the other fragment ions in the mass spectrum of **1** the situation is less clear and their formation will not be discussed here.

**Scheme 1:**
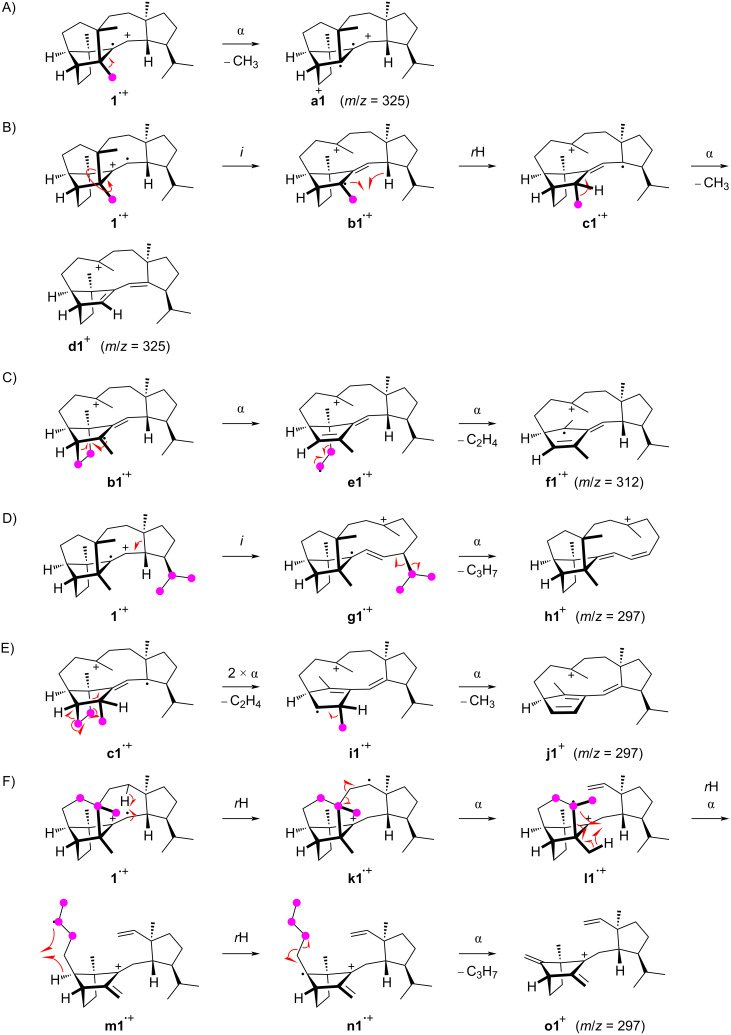
The EIMS fragmentation mechanisms for **1** explaining the formation of the fragment ions at *m*/*z* = 325, 312, and 297. Lost carbons are marked by purple dots.

The formation of the fragment ion at *m*/*z* = 325 requires the loss of one methyl group for which only C22, C23, C24, and C25, but not C20 and C21 show a significant participation. The most prominent loss is observed for C23 in an allylic position of the double bond in **1**. After electron impact ionisation preferentially at the π-system of the olefinic double bond the radical cation **1^•+^** is obtained from which the methyl group C23 can directly be lost by an α-cleavage leading to fragment **a1^+^** (Scheme 1A). However, the radical centred at the bridgehead carbon C11 is orthogonal to, or in other words, not in conjugation with the radical cation at C12–13. Therefore, an energetically more feasible process may be represented by an inductive cleavage leading to **b1^•+^**, a hydrogen rearrangement to **c1^•+^**, and an α-cleavage to **d1^+^** (Scheme 1B). The formation of the fragment ion at *m*/*z* = 312 proceeds through a highly specific loss of the C8–9 portion of **1**. This is explainable from **b1^•+^** by a sequence of two α-cleavages first to **e1^•+^** and then to **f1^•+^** with a neutral loss of ethylene (Scheme 1C). The fragment ion at *m*/*z* = 297 requires the loss of C_3_H_7_ which can be achieved by various reactions, as indicated by the PMA_297_. This may be realised by the cleavage of an intact C_3_H_7_ unit originating from the isopropyl group C20–19–21 or, by involving multiple C–C bond cleavages and hydrogen rearrangements, from the C25–3–4 portion. Alternatively, a combined loss of the C8–9 moiety and one methyl group (C22, C23, C24, or C25) is possible which basically combines the fragmentations of Scheme 1A and Scheme 1B. The loss of the isopropyl group C20–19–21 can be achieved by an inductive cleavage of **1^•+^** to **g1^•+^** followed by an α-cleavage to **h1^+^** (Scheme 1D). Starting from **c1^•+^**, two α-cleavages with the extrusion of ethylene can lead to **i1^•+^** that upon a third α-fragmentation with loss of the methyl group C23 results in **j1^+^** (Scheme 1E). The fragmentation of the C25–3–4 portion can be explained starting from **1^•+^** by a hydrogen rearrangement to **k1^•+^** and α-cleavage to **l1^•+^** (Scheme 1F). Another hydrogen rearrangement combined with an α-fragmentation then leads to the allyl cation **m1^•+^** which may undergo a third hydrogen rearrangement to **n1^•+^** and final cleavage of a propyl group to **o1^+^**.

**Scheme 2:**
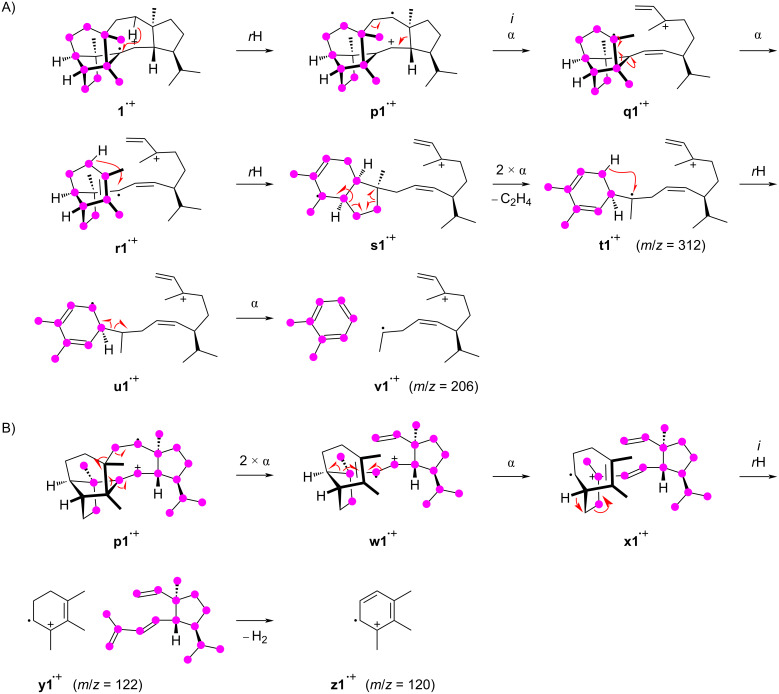
The EIMS fragmentation mechanisms for **1** explaining the formation of fragment ions at *m*/*z* = 206 and 120. Lost carbons are marked by purple dots.

The formation of the fragment ion at *m*/*z* = 206 proceeds with the loss of the portion represented by carbons C25–3–4–5–6–10(–9–8)–11–23 and can be proposed as shown in Scheme 2A. After the ionisation to **1^•+^** a hydrogen rearrangement leads to **p1^•+^** that further reacts by an inductive ring opening and α-cleavage to **q1^•+^**. Another α-fragmentation to **r1^•+^** may be followed by a hydrogen rearrangement to **s1^•+^** and two α-cleavages to **t1^•+^**, giving an alternative mechanistic explanation for the fragment ion at *m*/*z* = 312 by loss of C8–9. Another the hydrogen rearrangement to **u1^•+^** sets the stage for a final α-fragmentation with the neutral loss of *o*-xylene to **v1^•+^**. The base peak in the mass spectrum of **1** is formed from carbons C25–3–4–5–6–10(–9)–11–23, which can also be explained starting from **p1^•+^** by three sequential α-cleavages through **w1^•+^** to **x1^•+^** (Scheme 2B). The inductive cleavage with hydride migration leads to **y1^•+^** representing the minor fragment ion at *m*/*z* = 122 that may efficiently lose two hydrogens to give the conjugated system in **z1^•+^**.

### Fragmentation mechanisms for sestermobaraene B (**2**)

The position-specific mass shift analyses for sestermobaraene B (**2**) are based on the mass spectrum of the unlabelled compound in comparison to those of its 25 ^13^C-labelled isotopomers (Figure S2 in Supporting Information File 1). Clear results could be obtained for the fragment ions in the high mass region at *m*/*z* = 325, 312, and 297, for the base peak at *m*/*z* = 120, and the prominent fragment ion at *m*/*z* = 203. The results of the analyses are summarised in Figure 3.

**Scheme 3:**
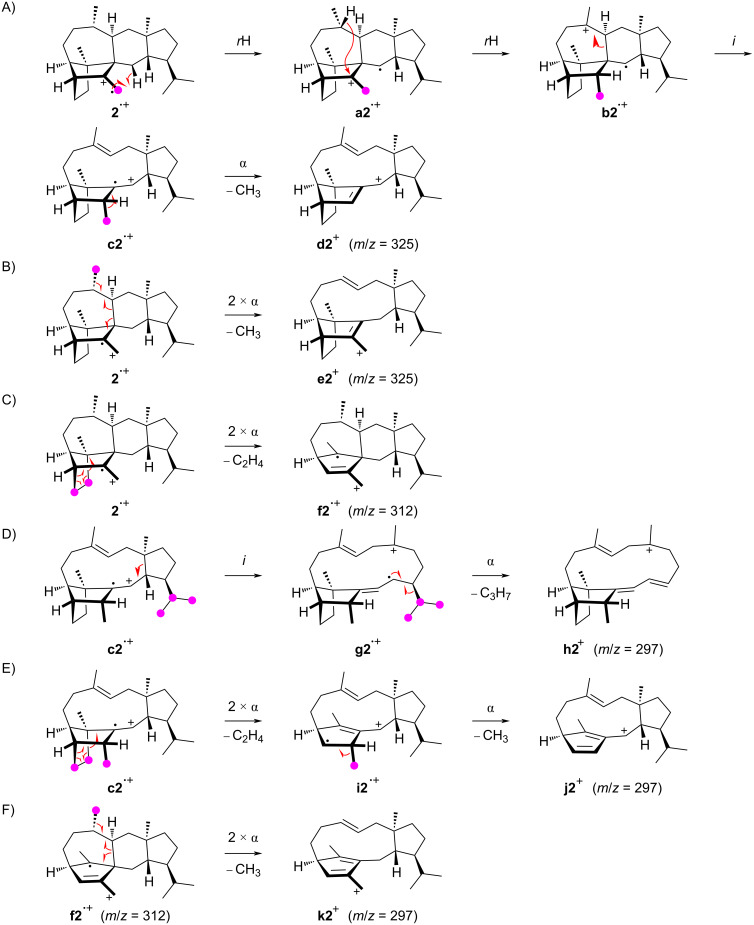
The EIMS fragmentation mechanisms for **2** explaining the formation of the fragment ions at *m*/*z* = 325, 312, and 297. Lost carbons are marked by purple dots.

Similarly to the observations made for **1**, also for **2** the formation of the fragment ion at *m*/*z* = 325 by loss of one methyl group proceeds by the cleavage of C22, C23, C24, or C25, while the fragmentation of C20 or C21 does not make a significant contribution. Notably, even from the olefinic methylene group C23 a methyl group can be cleaved off, which requires hydrogen rearrangements prior to the fragmentation. A possible mechanism starts from **2^•+^** by the hydrogen rearrangement to **a2^•+^** and a hydride shift to **b2^•+^** (Scheme 3A). This hydride migration is in reverse order compared to a similar step along the cationic cyclisation cascade during the biosynthesis of **2** (Scheme S1 in Supporting Information File 1). The subsequent inductive ring opening to **c2^•+^** and α-cleavage of C23 result in **d2^+^**. The losses of the other methyl groups can be understood more easily, e.g., two α-fragmentations from **2^•+^** explain the formation of **e2^+^** with the loss of C25 (Scheme 3B). The fragment ion at *m*/*z* = 312 arises by the loss of the C8–9 portion through a double α-cleavage from **2^•+^**, yielding to **f2^•+^** (Scheme 3C). Also for compound **2** different mechanisms for the formation of the fragment ion at *m*/*z* = 297 are observed, including the loss of the isopropyl group C20–19–21 or the loss of C8–9 and one methyl group. The cleavage of the isopropyl group is possible from **c2^•+^** by an inductive ring opening to **g2^•+^** and α-fragmentation to **h2^+^** (Scheme 3D). Alternatively, **c2^•+^** can react by two α-cleavages leading to **i2^•+^** with a neutral loss of ethylene, followed by another α-cleavage of C23 to **j2^+^** (Scheme 3E). The fragment ion at *m*/*z* = 297 can also be rationalised from **f2^•+^** by two α-fragmentations with the loss of C25 to result in **k2^+^** (Scheme 3F).

**Scheme 4:**
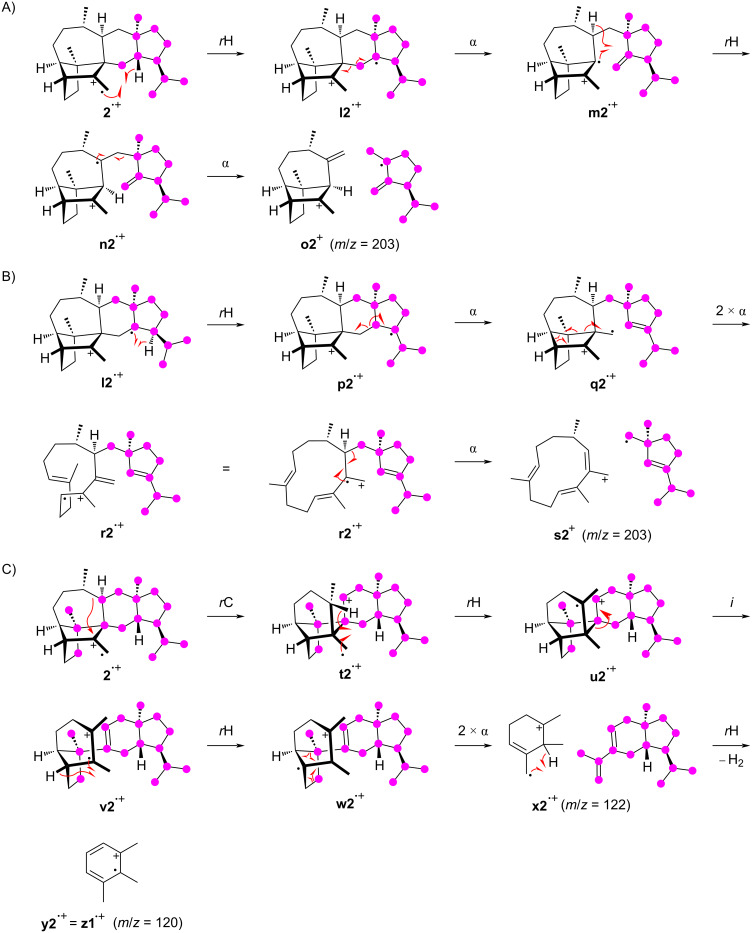
The EIMS fragmentation mechanisms for **2** explaining the formation of the fragment ions at *m*/*z* = 203 and 120. Lost carbons are marked by purple dots.

The position-specific mass shift analysis for *m*/*z* = 203 indicates the formation of this fragment ion by two overlaid mechanisms that both involve the loss of C14–15(–22)–16–17–18–19(–21)–20 plus either C13 or C1. A mechanistic model for the first case with loss of C13 starts from **2^•+^** by a hydrogen rearrangement to **l2^•+^** and an α-fragmentation to **m2^•+^**, followed by another hydrogen transfer to **n2^•+^** and α-cleavage to **o2^+^** (Scheme 4A). The second possibility with the loss of C1 is explainable from **l2^•+^** by a hydrogen migration to **p2^•+^** and an α-fragmentation to **q2^•+^**, followed by two more α-fragmentations to **r2^•+^** (Scheme 4B). A final α-cleavage then yields the target ion **s2^+^**. The generation of the base peak ion at *m*/*z* = 120 from the C25–3–4–5–6–10(–9)–11–23 moiety of **2** is more difficult to understand, as it must proceed with four C–C bond cleavages. Interestingly, for **2** the base peak is made up from the same portion of the molecule as for **1**, but while **1** has a bond between C3 and C11, this bond is missing in **2** that has a bond between C2 and C12 instead. For **1** the base peak was nicely explainable by the formation of an ionised aromatic ring system. In the first instance, it seems difficult to parallel this for **2**, but if for the first steps after ionisation to **2^•+^** a skeletal rearrangement to **t2^•+^** and a hydrogen transfer to **u2^•+^** are assumed, the parallelism of the fragmentation mechanisms becomes more obvious (Scheme 4C). Subsequent steps may include an inductive ring opening to **v2^•+^**, another hydrogen rearrangement to **w2^•+^**, and two α-cleavages to **x2^•+^**. Another hydrogen rearrangement and elimination of two hydrogen atoms lead to **y2^•+^** which is identical to **z1^•+^** in the fragmentation mechanism for the base peak ion of **1**.

### Fragmentation mechanisms for sestermobaraene C (**3**)

For sestermobaraene C (**3**) the position-specific mass shift analyses based on the mass spectra of the unlabelled versus all 25 isotopomers of the singly ^13^C-labelled material (Figure S3 in Supporting Information File 1) also gave unambiguous results for the fragment ions at *m*/*z* = 325, 312, 297, 206, and the base peak at *m*/*z* = 120 (Figure 4), which is similar to the corresponding analyses for **1** and **2** not only in the nominal masses of the fragment ions, but also in terms of the portions of the carbon skeletons these fragments arise from. Thus, it can be expected that similar fragmentation reactions as discussed for **1** and **2** above can lead to their formation. One notable difference is observed for the fragment ions at *m*/*z* = 312 and 297 that are formed with a partial loss of C11–23, which was not observed for compounds **1** and **2**.

**Scheme 5:**
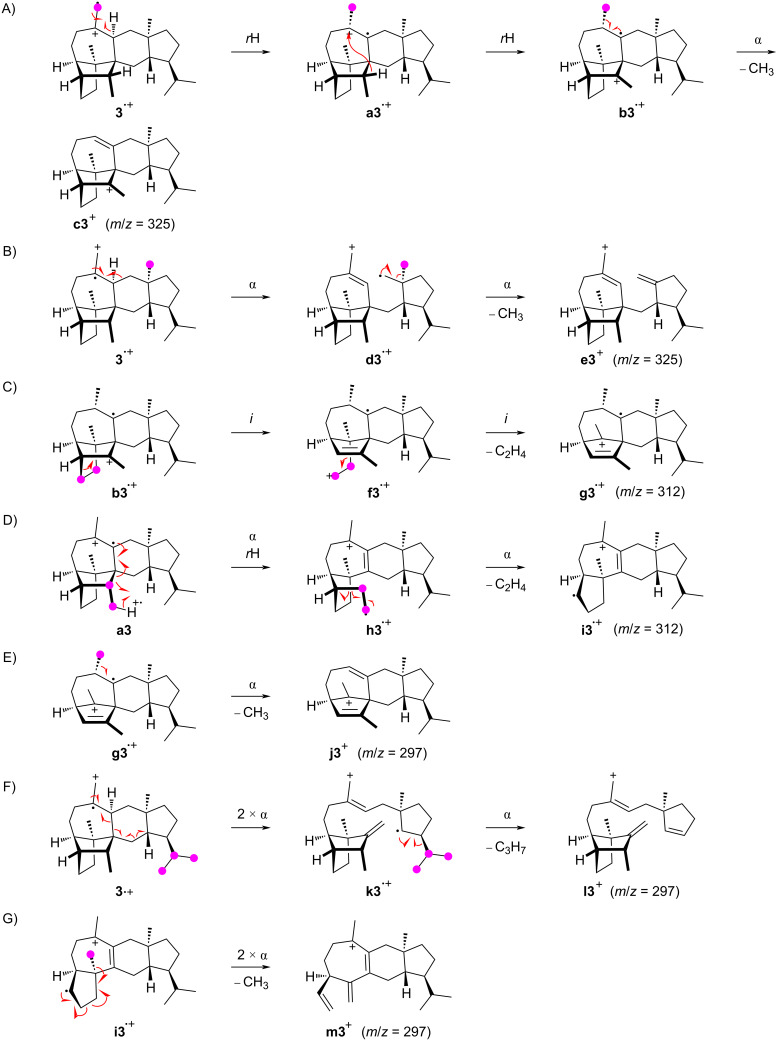
The EIMS fragmentation mechanisms for **3** explaining the formation of the fragment ions at *m*/*z* = 325, 312, and 297. Lost carbons are marked by purple dots.

The formation of the fragment ion at *m*/*z* = 325 proceeds with cleavage of C22, C23, C24, or C25, as observed before for compounds **1** and **2**. Especially noteworthy is the cleavage of the methylene carbon C25, which is explainable from **3^•+^** by a hydrogen rearrangement to **a3^•+^**, followed by a hydride shift to **b3^•+^** and an α-fragmentation to **c3^+^** (Scheme 5A). The alternative loss of C22 is possible from **3^•+^** by two sequential α-cleavages via **d3^•+^** to **e3^+^** (Scheme 5B). The fragment ion at *m*/*z* = 312 involves the loss of either the C8–9 or the C11–23 portion. The first case can be understood starting from **b3^•+^** by two inductive cleavages with the neutral loss of ethylene to **f3^•+^** and then **g3^•+^** (Scheme 5C), while the second case may start from **a3^•+^** by an α-cleavage with hydrogen rearrangement to **h3^•+^** and another subsequent α-fragmentation to **i3^•+^** (Scheme 5D). Similar to the observations for compounds **1** and **2**, the fragment ion at *m*/*z* = 297 of **3** is generated by the loss of C8–9 and one methyl group or of the isopropyl group C20–19–21. In addition, the combined loss of C11–23 and one methyl group also contributes to its formation. The possible mechanistic models include a simple α-fragmentation with the loss of C25 from **g3^•+^** to **j3^+^** (Scheme 5E), a sequence of three α-cleavages from **3^•+^** through **k3^•+^** leading to **l3^+^** (Scheme 5F), and a double α-fragmentation in **i3^•+^** that explains the formation of **m3^+^** (Scheme 5G).

**Scheme 6:**
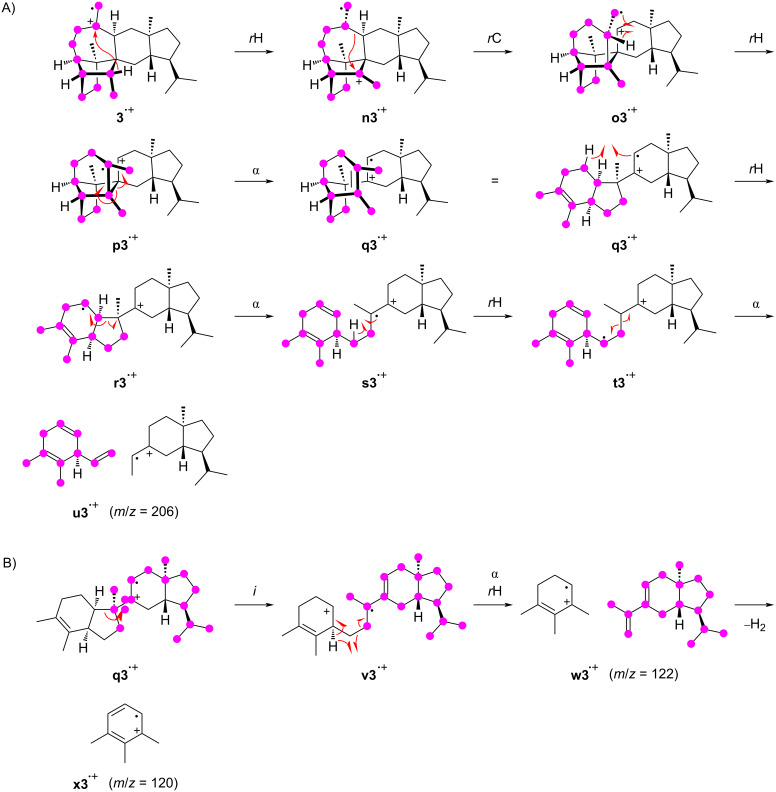
The EIMS fragmentation mechanisms for **3** explaining the formation of the fragment ion at *m*/*z* = 206 and the base peak ion at *m*/*z* = 120. Lost carbons are marked by purple dots.

The fragment ion at *m*/*z* = 206 arises from the C25–3–4–5–6–10(–9–8)–11–23 moiety of **3**. Its formation requires multiple bond cleavages and hydrogen transfers and is thus a multistep process (Scheme 6A). Starting from **3^•+^**, a hydride shift to **n3^•+^** and skeletal rearrangement lead to **o3^•+^**. A subsequent hydrogen rearrangement of this primary radical yields the tertiary radical **p3^•+^** that can undergo an α-fragmentation to **q3^•+^**, followed by hydrogen rearrangement to **r3^•+^**, setting the stage for the next α-cleavage to **s3^•+^**. The same principle can explain the last bond cleavage: A hydride shift to **t3^•+^** adjusts the reactivity for the α-fragmentation to **u3^•+^**. Notably, the intermediate **q3^•+^** is also a good starting point to explain the formation of the base peak ion at *m*/*z* = 120 (Scheme 6B). The inductive ring opening produces **v3^•+^** that, upon α-cleavage with hydrogen rearrangement, leads to **w3^•+^** (*m*/*z* = 122). The base peak ion **x3^•+^** then results by the loss of two hydrogens.

## Conclusion

In this work we demonstrated that ^13^C-labellings can efficiently be introduced by terpene synthase catalysed reactions into each single position of a terpene, which is useful for the deep investigations on mass spectrometric fragmentation reactions. The present study provides the first example for such investigations on sesterterpene fragmentations. The applied method, once the synthetic ^13^C-labelled oligoprenyl diphosphates are at hand, is superior to any other approach for the introduction of labellings, also because the labelled terpene precursors can be used for studies on many different terpenes for which terpene synthases are available. In the present case it is intriguing to learn that, although the structures of the three investigated sesterterpenes are different, not only similar fragment ions are observed, but also similar reactions lead to their formation, which is most prominently observed for the common base peak ion at *m*/*z* = 120 for all three compounds. This means that the sesterterpenes have a common intrinsic reactivity that is in the first instance reflected by their joint biosynthesis, but also by their similar behaviour in the comparably high-energy chemistry of mass spectrometric fragmentation reactions. Further support for the similar reactivity of the investigated compounds during biosynthesis and mass spectrometric fragmentations is given by the notable observation of hydride shifts that occur in both of these processes. However, the three compounds show also some differences in their mass spectrometric fragmentation, e.g., for compound **2** a strong fragment ion is observed at *m*/*z* = 203, which is much less relevant for the other two compounds. It should be emphasised that the mechanistic hypotheses presented in this work are solely based on the ^13^C-labellings, while specific hydrogen migrations would need to be followed by deuterium labellings, but in these cases data interpretation may be hampered by kinetic isotope effects. Nevertheless, at the current stage it cannot be excluded that such experiments could demonstrate the need for a refinement of the fragmentation mechanisms for certain fragment ions presented here. We will continue our investigations on terpene fragmentations in EIMS in the future by the strategy applied in this work to learn more about the underlying reaction mechanisms.

## Experimental

### Preparation of ^13^C-labelled compounds **1**–**3** and GC–MS analysis

The 25 isotopomers of (^13^C)-**1**, (^13^C)-**2**, and (^13^C)-**3** were prepared enzymatically with SmTS1 from the correspondingly labelled oligoprenyl diphosphates as reported previously [1]. The compounds were obtained as mixtures that were directly analysed by GC–MS. The GC–MS analyses were performed using a 7890A GC connected to a 5977A mass selective detector (Agilent, Hewlett-Packard Company, Wilmington, USA). The gas chromatographic separation was done using a HP5-MS fused silica capillary column (30 m, 0.25 mm i.d., 0.25 μm film, Agilent). The GC settings were 1) inlet pressure: 77.1 kPa, He 23.3 mL min^−1^; 2) injector temperature: 250 °C; 3) injection volume: 2 μL; 4) injector operation mode: splitless (60 s valve time); 5) carrier gas: He at 1.2 mL min^−1^; 6) temperature program: 5 min at 50 °C, then increasing with a ramp of 5 °C min^−1^ to 320 °C. The MS settings were 1) transfer line: 300 °C; 2) electron energy: 70 eV.

## Supporting Information

File 1 Mass spectra of the unlabelled and ^13^C-labelled compounds **1**–**3**, and the cyclisation mechanism from GFPP to **1**–**3** by SmTS1.
