# Supplementary material for: On the mass spectrometric fragmentations of the bacterial sesterterpenes sestermobaraenes A–C
Source: Beilstein J Org Chem. 2020 Nov 19;16:2807–19. doi: 10.3762/bjoc.16.231 (PMC7684692; doi:10.3762/bjoc.16.231)
Supplement: File 1 — Mass spectra of the unlabelled and 13C-labelled compounds 1–3, and the cyclisation mechanism from GFPP to 1–3 by SmTS1. [file Beilstein_J_Org_Chem-16-2807-s001.pdf]

BEILSTEIN JOURNAL OF ORGANIC CHEMISTRY

Supporting Information

for

On the mass spectrometric fragmentations of the bacterial sesterterpenes sestermobaraenes A–C

Anwei Hou and Jeroen S. Dickschat

Beilstein J. Org. Chem. **2020,** *16,* 2807–2819. doi:10.3762/bjoc.16.231

Mass spectra of the unlabelled and ^{13}C-labelled compounds 1–3, and the cyclisation mechanism from GFPP to 1–3 by SmTS1

License and Terms: This is a supporting information file under the terms of the Creative Commons Attribution License (https://creativecommons.org/licenses/by/4.0). Please note that the reuse, redistribution and reproduction in particular requires that the authors and source are credited.
The license is subject to the *Beilstein Journal of Organic Chemistry* terms and conditions: (https://www.beilstein-journals.org/bjoc)

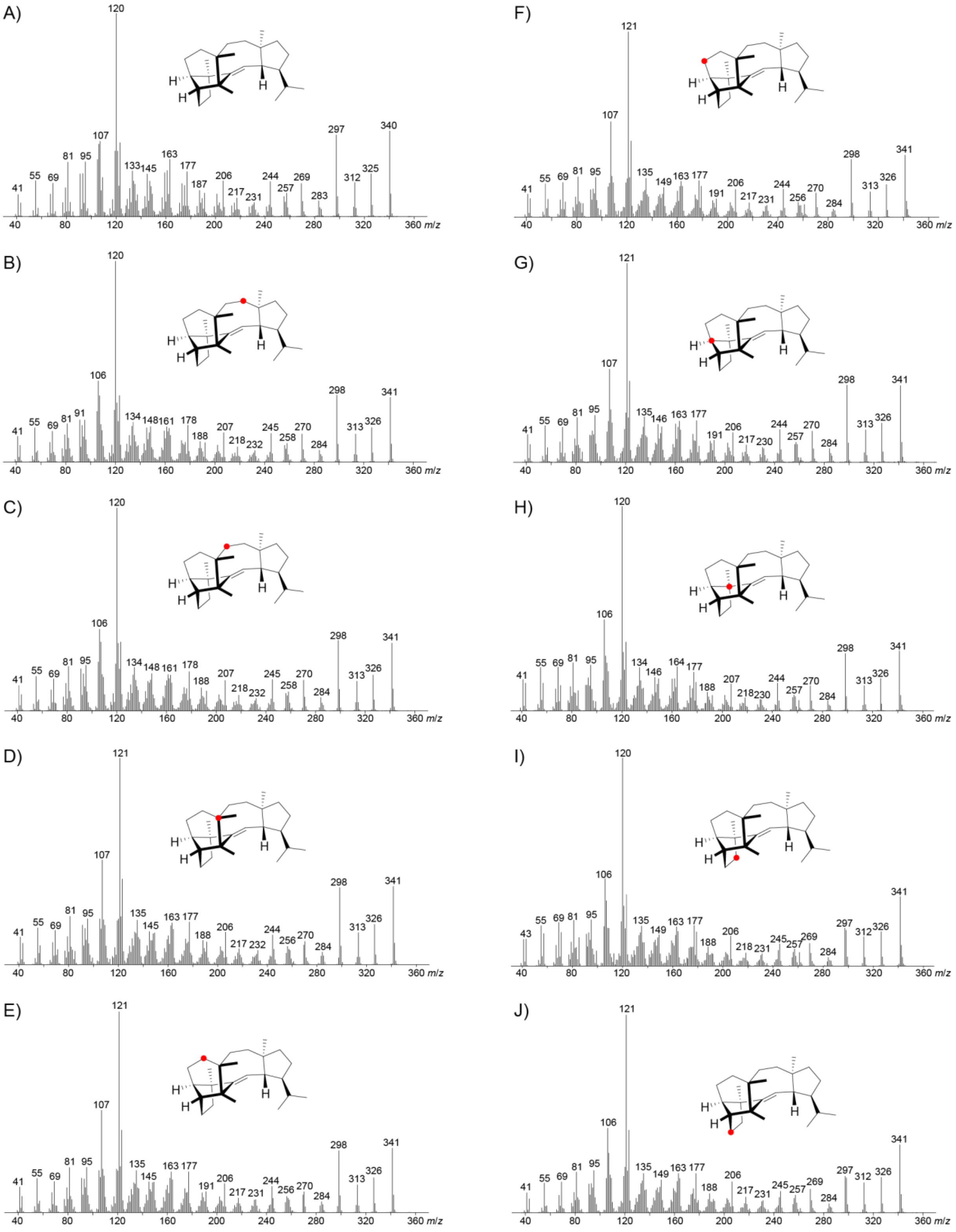

Figure S1: EI mass spectra of A) unlabelled **1** and of B)–J) (^{13}C)-**1** with labellings at carbons C1–C9. Red dots indicate ^{13}C-labelled carbons.

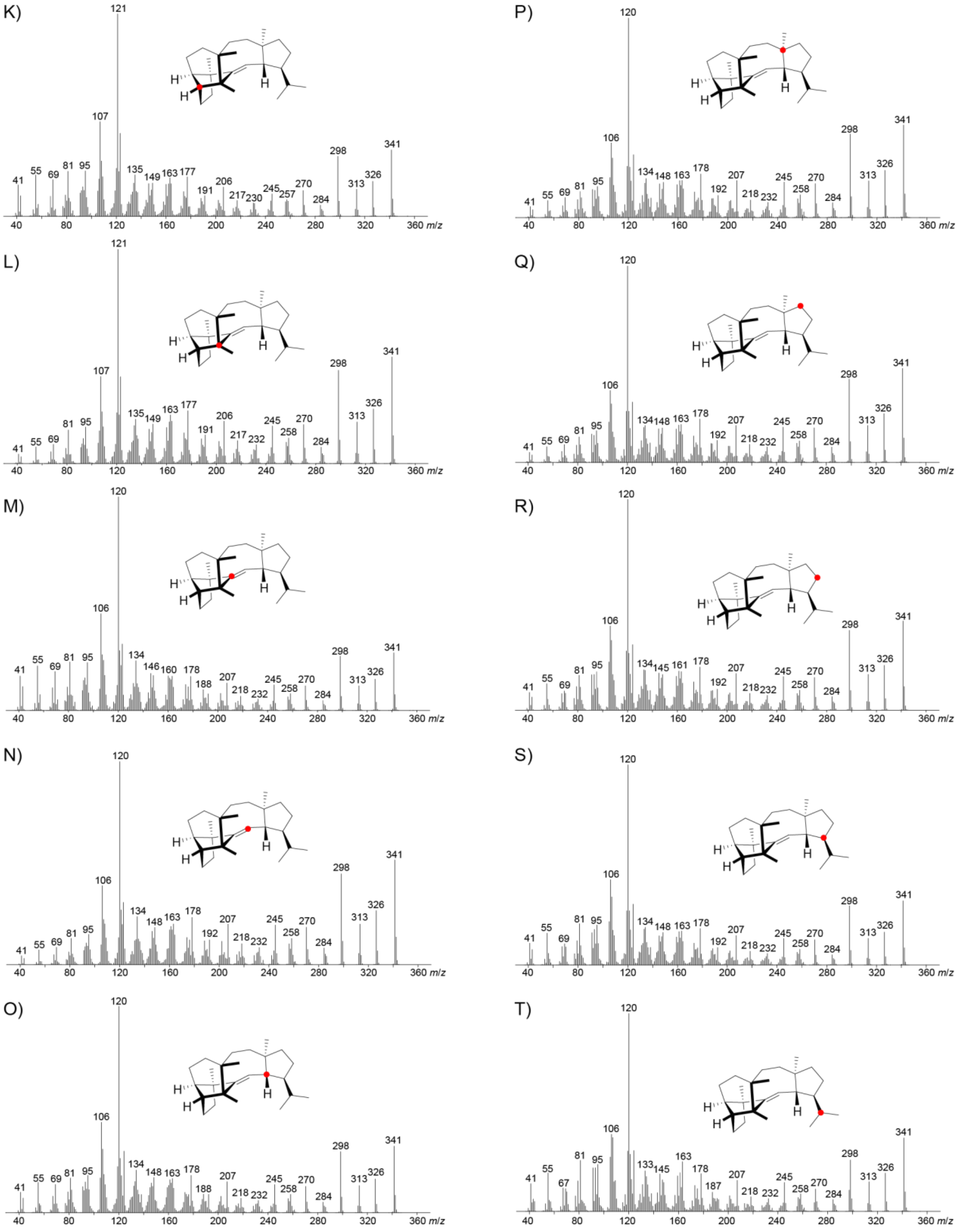

Figure S1 (continued): EI mass spectra of K)–T) (^{13}C)-**1** with labellings at carbons C10–C19. Red dots indicate ^{13}C-labelled carbons.

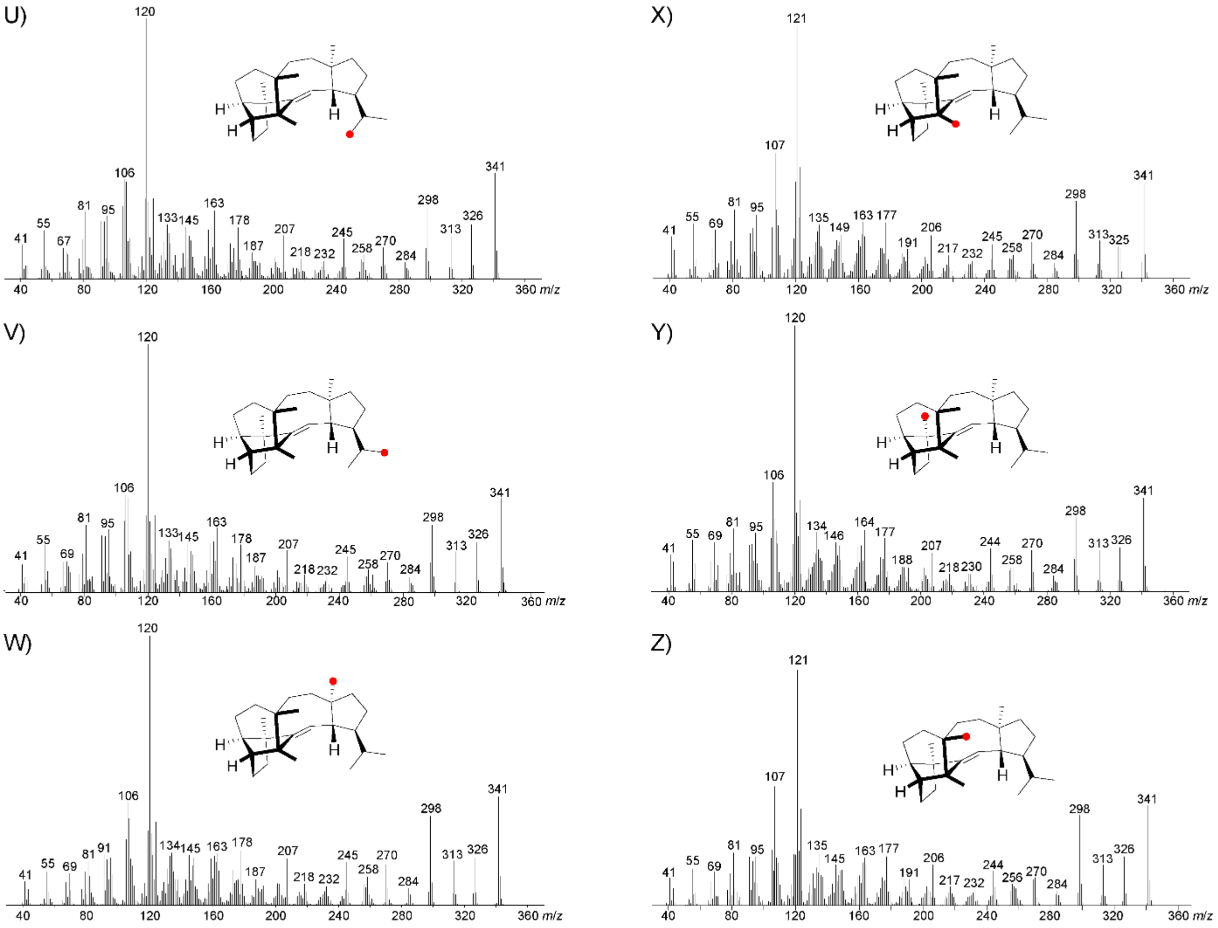

Figure S1 (continued): EI mass spectra of U)–Z) (^{13}C)-**1** with labellings at carbons C20–C25. Red dots indicate ^{13}C-labelled carbons.

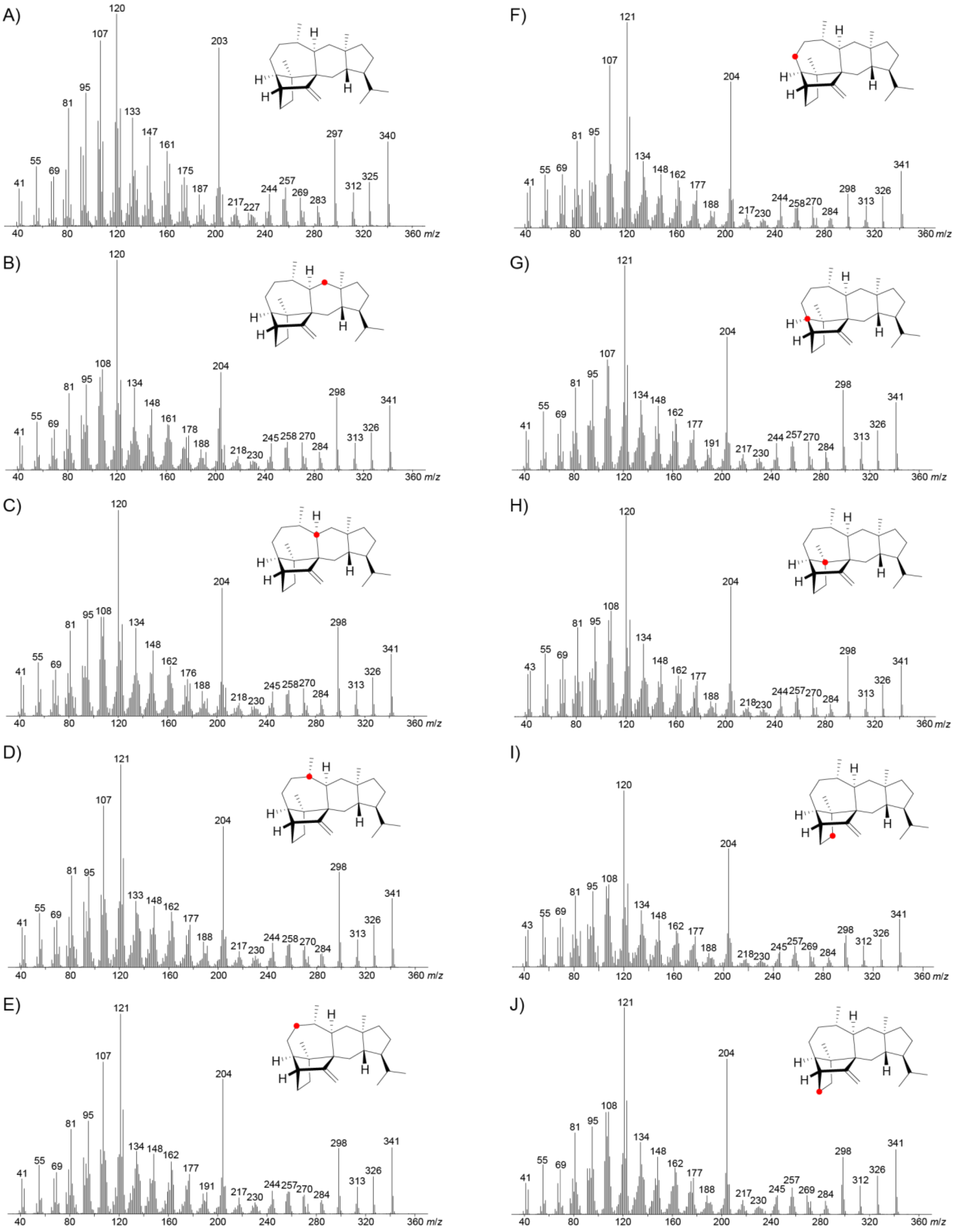

Figure S2: EI mass spectra of A) unlabelled **2** and of B)–J) (^{13}C)-**2** with labellings at carbons C1–C9. Red dots indicate ^{13}C-labelled carbons.

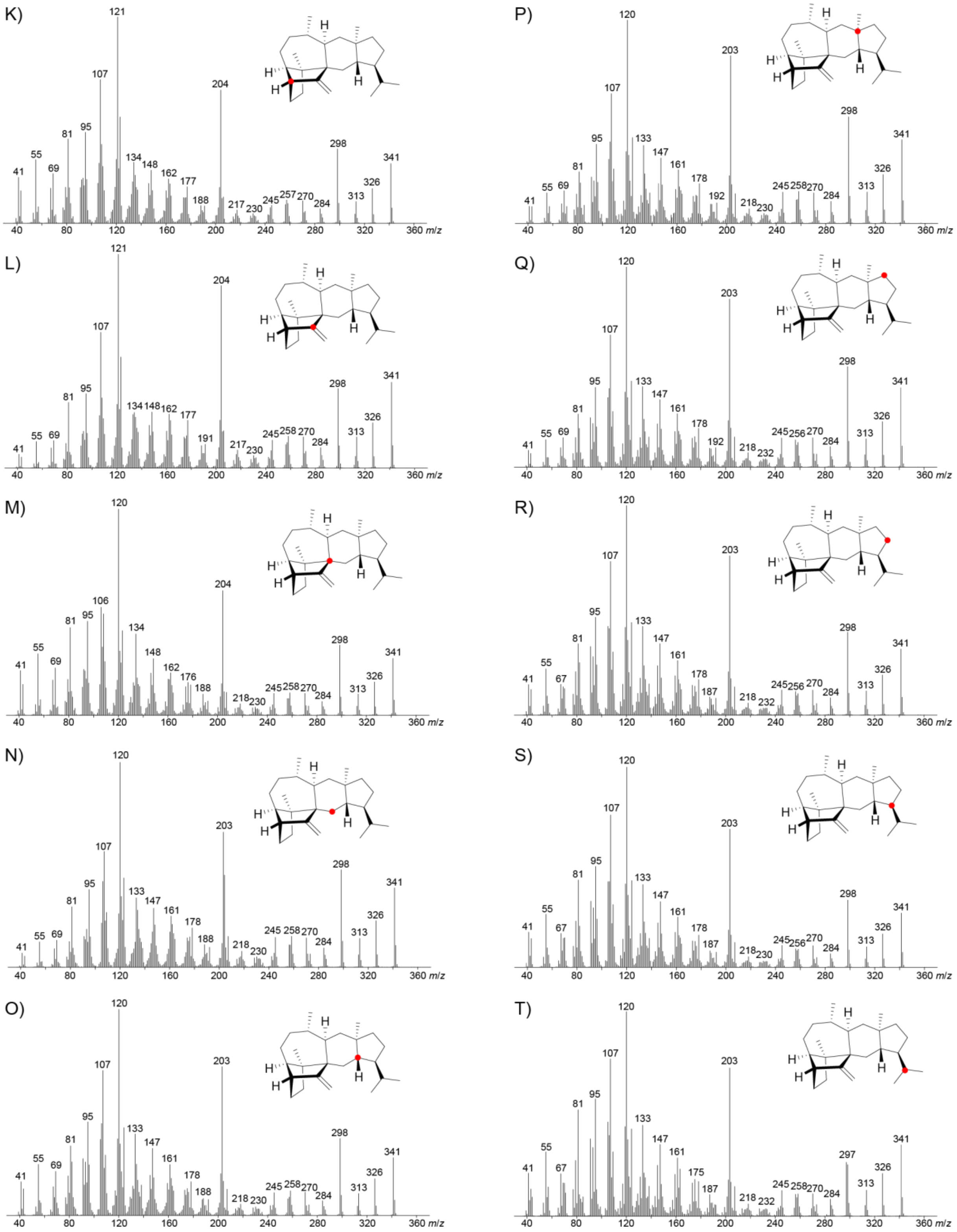

Figure S2 (continued): EI mass spectra of K)–T) (^{13}C)-**2** with labellings at carbons C10–C19. Red dots indicate ^{13}C-labelled carbons.

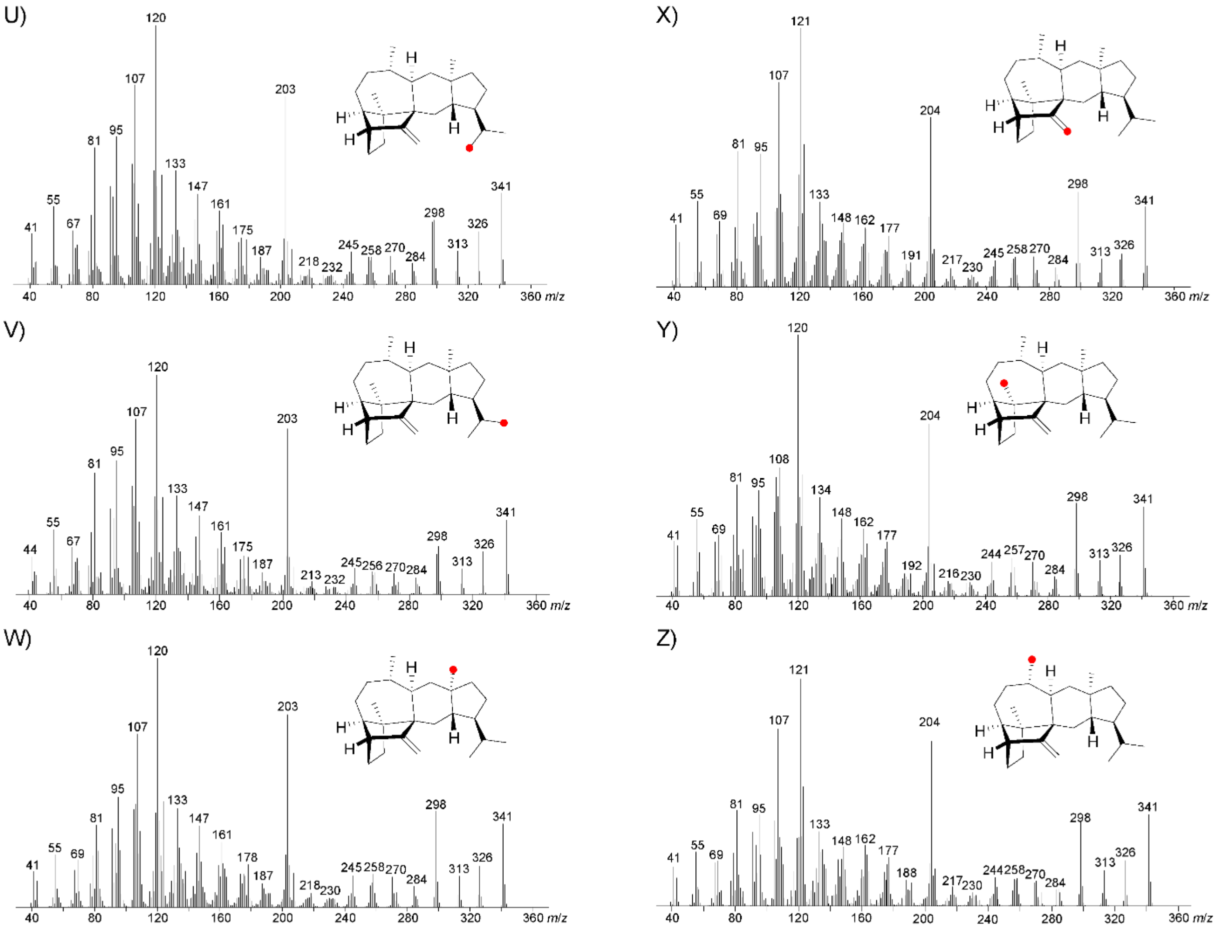

Figure S2 (continued): EI mass spectra of U)–Z) (^{13}C)-**2** with labellings at carbons C20–C25. Red dots indicate ^{13}C-labelled carbons.

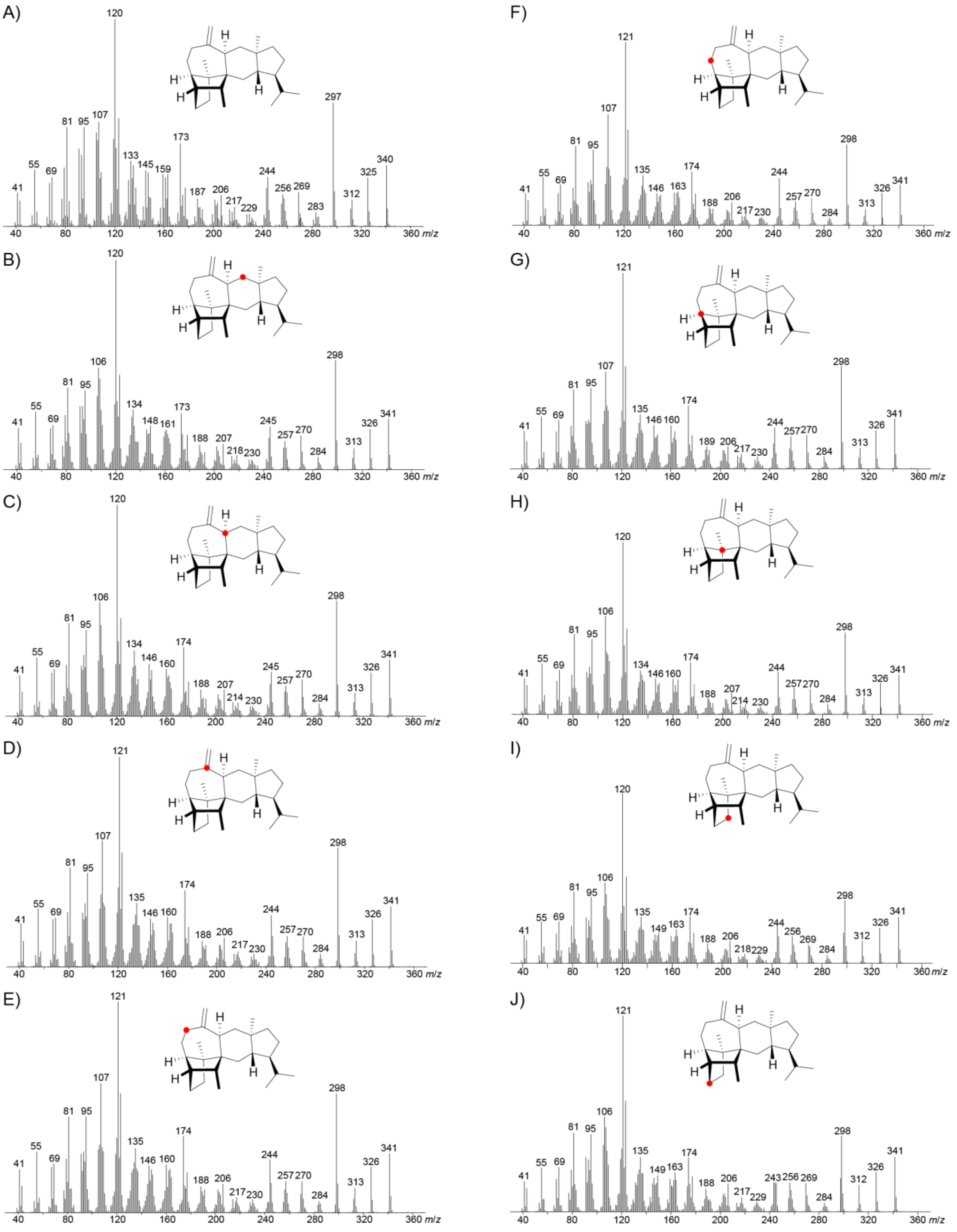

Figure S3: EI mass spectra of A) unlabelled **3** and of B)–J) (^{13}C)-**1** with labellings at carbons C1–C9. Red dots indicate ^{13}C-labelled carbons.

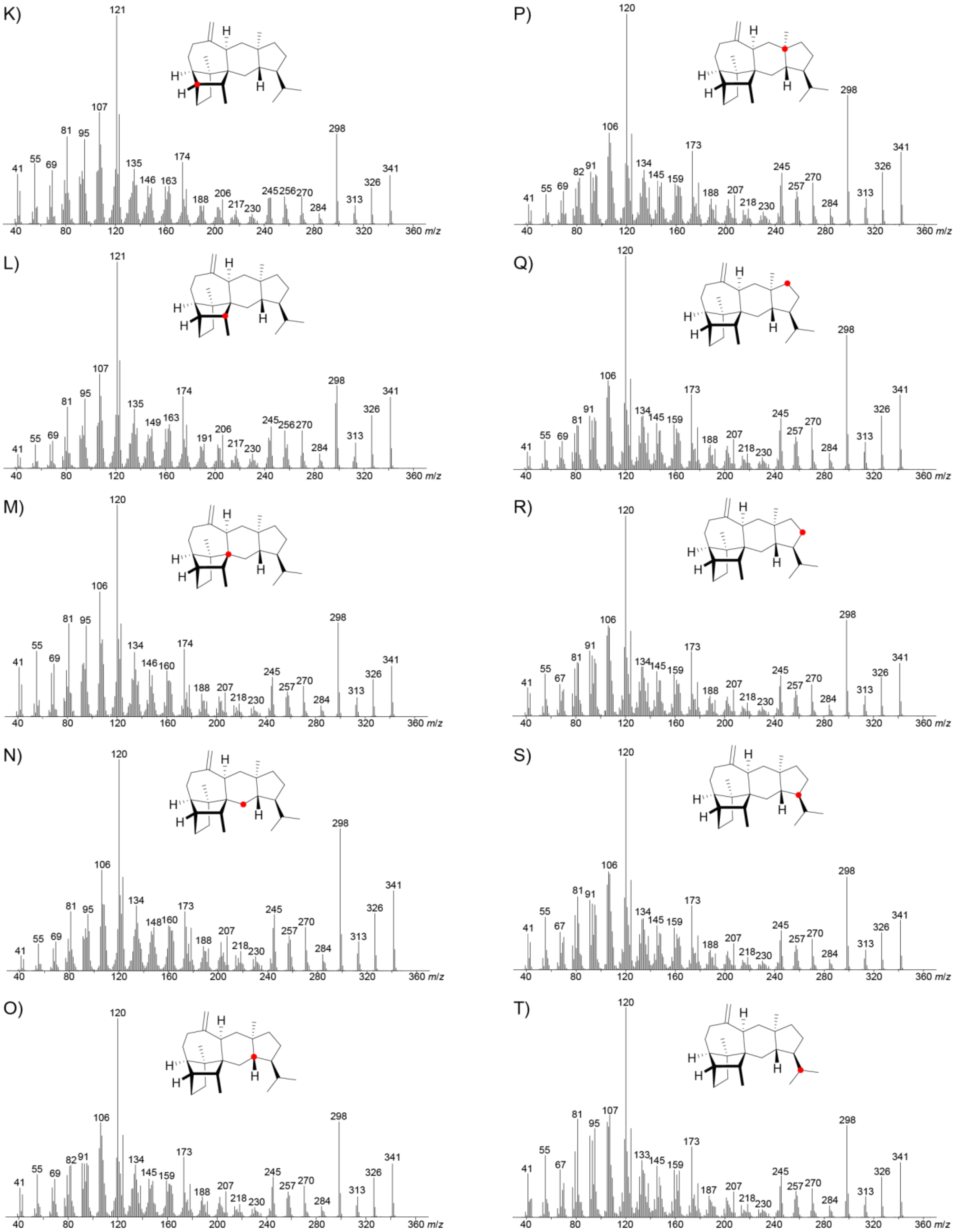

Figure S3 (continued): EI mass spectra of K)–T) (^{13}C)-**3** with labellings at carbons C10–C19. Red dots indicate ^{13}C-labelled carbons.

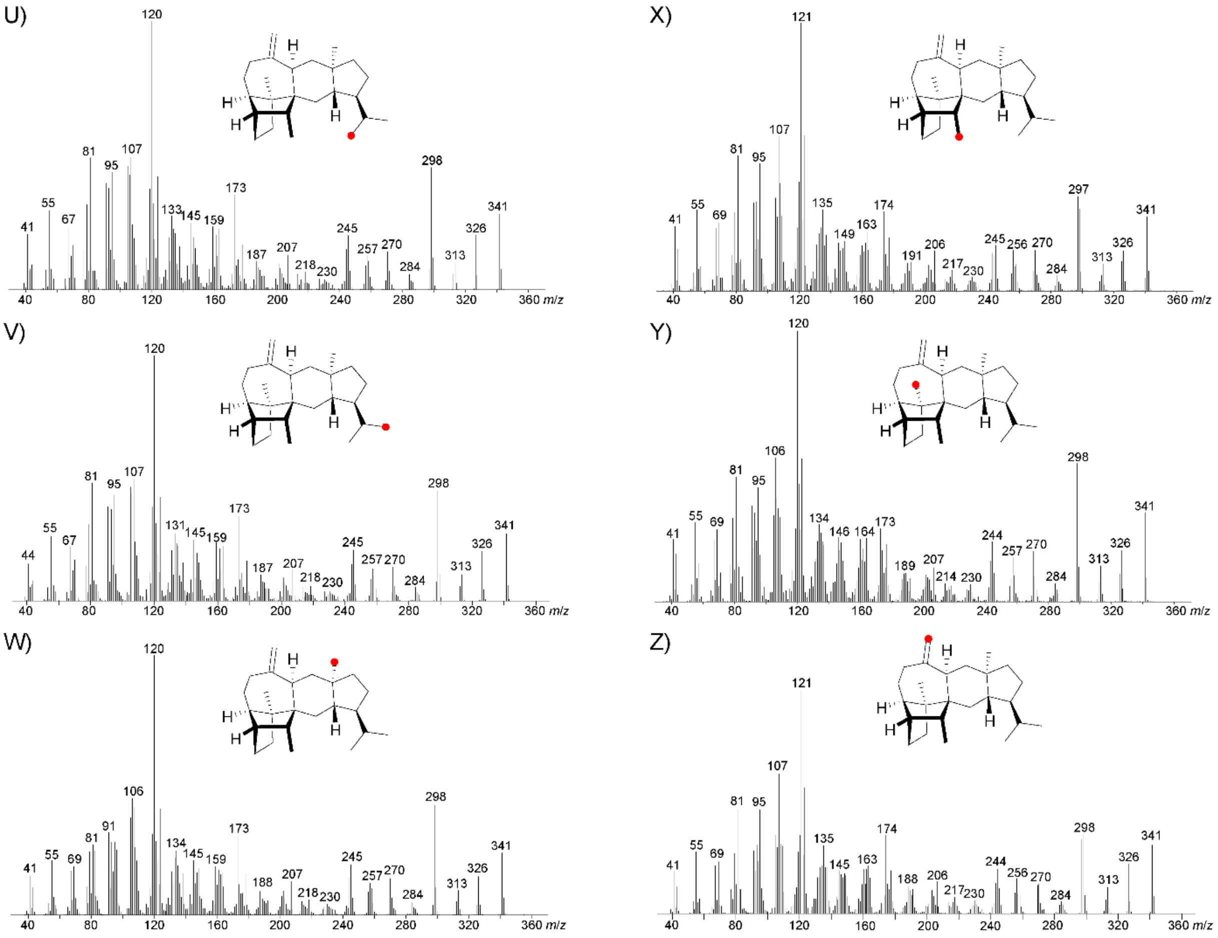

Figure S3 (continued): EI mass spectra of U)–Z) (^{13}C)-**3** with labellings at carbons C20–C25. Red dots indicate ^{13}C-labelled carbons.

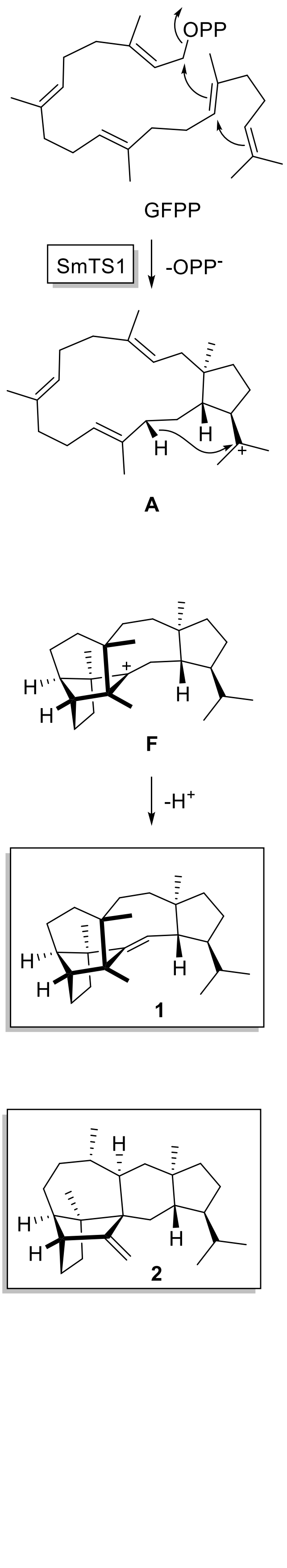

Scheme S1: Cyclisation mechanism from GFPP to **1–3** by SmTS1.
